# New Genotype of *Coxiella burnetii* Causing Epizootic Q Fever Outbreak in Rodents, Northern Senegal

**DOI:** 10.3201/eid2905.221034

**Published:** 2023-05

**Authors:** Joa Mangombi-Pambou, Laurent Granjon, Clément Labarrere, Mamadou Kane, Youssoupha Niang, Pierre-Edouard Fournier, Jérémy Delerce, Florence Fenollar, Oleg Mediannikov

**Affiliations:** Centre Interdisciplinaire de Recherches Médicales de Franceville, Franceville, Gabon (J. Mangombi-Pambou);; Aix-Marseille University, Marseille, France (J. Mangombi-Pambou, C. Labarrere, P.-E. Fournier, J. Delerce, F. Fenollar, O. Mediannikov);; University Hospital Institute Méditerranée Infection, Marseille (J. Mangombi-Pambou, C. Labarrere, P.-E. Fournier, F. Fenollar, O. Mediannikov);; Centre de Biologie pour la Gestion des Populations, Montpellier, France (L. Granjon);; Biologie des Populations Animales Sahelo-Soudaniennes, Dakar, Senegal (M. Kane, Y. Niang)

**Keywords:** *Coxiella burnetii*, Q fever, bacteria, airborne diseases, rodents, Senegal, zoonoses

## Abstract

In Senegal, *Coxiella burnetii*, which causes Q fever, has often been identified in ticks and humans near livestock, which are considered to be reservoirs and main sources of infection. We describe the emergence of *C. burnetii* in rodents, not previously known to carry this pathogen, and describe 2 new genotypes.

*Coxiella burnetii* is a causative agent of Q fever, a worldwide zoonosis. The disease may be acute (relatively benign) or chronic (with a wide range of clinical manifestations that can lead to high human mortality) (*1*). Humans are infected by inhaling contaminated environmental dust and aerosol particles from the birth products of infected animals, as well as through direct contact with milk, urine, or feces containing *C. burnetii* (*2*,*3*). Humans are not considered natural hosts of *C. burnetii* (*4*). A wide spectrum of animals can serve as hosts (*4*), but the reservoirs are livestock, mainly sheep, cattle, and goats (*3*), which are also the main sources of human infection (*1*). 

In Senegal, *C. burnetii* has been reported in humans (*4*–*6*) and ticks (*4*). It has been isolated from rodent-associated soft ticks (*Ornithodoros sonrai*) and detected in several species of hard ticks collected from ruminants (*4*). Our previous study of zoonotic pathogens in rodents collected in 2017 revealed no presence of *C. burnetii* in rodent populations from the Ferlo region in northern Senegal (*7*). However, in this study, we tested rodent samples collected during 2019–2020 from the same region and found high prevalence of a new *C. burnetii* genotype, which might indicate an ongoing epizootic outbreak. 

We screened 125 rodent samples for *C. burnetii*; the rodents were collected in the Ferlo region in northern Senegal near Widou Thiengoly (15.99°N, 15.32°W) under framework agreements between the French National Research Institute for Development and Senegal (*7*). None of the rodent species investigated were listed as protected with the International Union for Conservation of Nature or the Convention on International Trade in Endangered Species of Wild Fauna and Flora. Handling procedures were performed under Centre de Biologie Pour la Gestion des Populations agreement no. D-34-169-1 for experiments on wild animals and followed the guidelines of the American Society of Mammologists (*8*). 

Rodents sampled belonged to the species *Arvicanthis niloticus* (n = 29), *Desmodilliscus braueri* (n = 3), *Gerbillus nancillus* (n = 9), *G. nigeriae* (n = 71), *Jaculus jaculus* (n = 4), *Taterillus* spp. (probably *T. pygargus*) (n = 8), and *Xerus erythropus* (n = 1). We extracted DNA from the spleen as described elsewhere (*7*) and stored it at −20°C. We detected bacterial DNA using *C. burnetii*–specific quantitative real-time PCR with primers and probes targeting IS1111 and IS30A spacers (*4*). For positive samples with a cycle threshold value <38, we first amplified 3 pairs of intergenic spacer primers, Cox2F/R, Cox5F/R, and Cox18F/R (*5*). Multispacer sequence typing (MST) genotyping of *C. burnetii* strains using sequences from the amplification of these 3 primer pairs revealed a potential new genotype. We amplified the other 7 primer pairs, Cox20F/R, Cox22F/R, Cox37F/R, Cox51F/R, Cox56F/R, Cox57F/R, and Cox61F/R, to describe this genotype.

Overall, 22.4% (28/125 for IS1111) and 19.2% (24/125 for IS30A) of rodents screened were positive for *C. burnetii*–specific quantitative PCR: *Desmodilliscus braueri* (33.3%; 1/3), *G. nancillus* (33.3%; 3/9), *G. nigeriae* (28.2%; 20/71), *Jaculus* *jaculus* (25%; 1/4), and *Taterillus* spp. (37.5%, 3/8). We found no *Arvicanthis niloticus* or *Xerus erythropus *rodents positive for *C. burnetii*. We performed complete MST genotyping of positive* C. burnetii* strain samples, and the sequences obtained from the primer pairs showed 100% identity for all positive samples. Nevertheless, 1 sample showed an insertion of 5 nucleotide bases in the amplified sequence of Cox56 spacer (Table), indicating the presence of a probable variant. All of these sequences demonstrated the presence of >1 new genotypes of *C. burnetii* according to the BLAST database (https://ifr48.timone.univ-mrs.fr/mst/coxiellaburnetii/blast.html). Phylogenetic analysis based on concatenated sequences of spacers revealed that the new genotypes, MST75 (major) and MST76 (with an insertion), are close together and cluster with other genotypes, including those already found in Senegal, such as MST19, and those infecting animals and humans, such as MST16, MST17, MST20, and MST61 (Figure). 

**Table:** Characterization of new *Coxiella burnetii* MST75 and MST 76 genotypes described in study of new genotype of *C. burnetii* causing epizootic Q fever outbreak in rodents, northern Senegal*

Species	No.	Is1111 positive, no. (%)	Profile of spacers										Genotype
			Cox2	Cox5	Cox18	Cox20	Cox22	Cox37	Cox51	Cox56	Cox57	Cox61	
*Arvicanthis niloticus*	29	NA	NA	NA	NA	NA	NA	NA	NA	NA	NA	NA	NA
*Desmodilliscus braueri*	3	1 (33.3)	3	14	6	6	5	4	10	10	13	5	MST75
*Gerbillus nancillus*	9	3 (33.3)	3	14	6	6	5	4	10	10	13	5	MST75
*G. nigeriae*†	71	20 (28.2)	3	14	6	6	5	4	10	20/21‡	13	5	MST75/ MST76‡
*Jaculus jaculus*	4	1 (25)	3	14	6	6	5	4	10	10	13	5	MST75
*Taterillus* spp.	8	3 (37.5)	3	14	6	6	5	.4	10	10	13	5	MST75
*Xerus erythropus*	1	NA	NA	NA	NA	NA	NA	NA	NA	NA	NA	NA	NA
Total	125	28	NA	NA	NA	NA	NA	NA	NA	NA	NA	NA	NA

*MST, multispacer sequence typing; NA, not applicable. †Invasive (expanding) species. ‡Genotype MST76 has the same profile as MST75 except for Cox 56.

**Figure:**
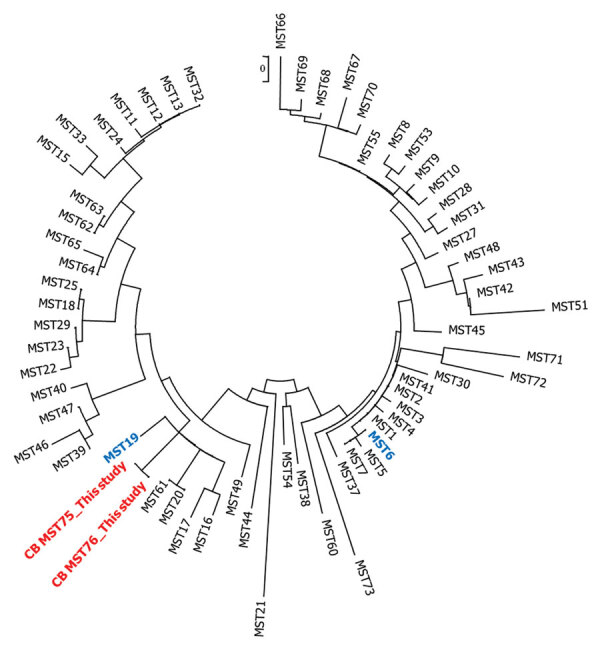
Neighbor-joining circular unrooted tree showing the relationship between *Coxiella burnetii* genotypes described in study of new genotype of *C. burnetii* causing epizootic Q fever outbreak in rodents, northern Senegal. MST75 and MST76 (red) were compared with genotypes already found in Senegal, MST19 and MST6 (blue), and other genotypes. The analysis involved 64 nt sequences. All positions containing gaps and missing data were eliminated. There were a total of 4,692 positions in the final dataset. Evolutionary analyses were conducted in MEGA7 (https://www.megasoftware.net). MST, multispacer sequence typing.

Our finding of *C. burnetii* MST75 and MST76 genotypes in the Ferlo rodent community suggests the emergence of a Q fever epizootic outbreak. Previously identified *C. burnetii* strains in Senegal were related to the proximity of livestock near the villages of Dielmo and Ndiop (*9*). In Ferlo, a previous study conducted on rodents sampled in 2017 did not find *C. burnetii* (*7*), indicating a relatively recent, possibly still ongoing epizootic outbreak. High *C. burnetii* prevalences (28%–38%) were observed in different species of gerbilline rodents, including *G. nigeriae*, which has recently colonized northern Senegal and is now the dominant species in outdoor rodent communities of Ferlo (*10*). The possibility of animal transmission from farms located near the rodent sampling area should also be explored. Our study shows the emergence in Senegal of new *C. burnetii* genotypes in susceptible animals, such as rodents (*1*), which might be a source of human infections. Although the pathogenicity of these new genotypes for humans is yet unknown, our findings signal the urgent need for epidemiologic surveillance for *C. burnetii *infection in humans in Senegal and neighboring countries. 
